# Validation of the extended version of the Implementation Leadership Scale (ILS-X)

**DOI:** 10.1186/s43058-025-00786-x

**Published:** 2025-12-04

**Authors:** Mark G. Ehrhart, Nathaniel J. Williams, Marisa Sklar, Nallely R. Vega, Alexandra Kandah, Gregory A. Aarons

**Affiliations:** 1 Department of Psychology, University of Central Florida, 4111 Pictor Lane, Orlando, FL 32816-1390 USA; 2 Institute for the Study of Behavioral Health and Addiction, Boise State University, 1910 University Drive, Education Suite 717, Boise, ID 83725-1940 USA; 3 School of Social Work, Boise State University, 1910 University Drive, Education Suite 717, Boise, ID 83725-1940 USA; 4 Department of Psychiatry, University of California, San Diego, 9500 Gilman Drive (0812), La Jolla, CA 92093-0812 USA; 5 UC San Diego ACTRI Dissemination and Implementation Science Center, University of California, San Diego, 9500 Gilman Drive, La Jolla, CA 92093 USA; 6 Child and Adolescent Services Research Center, 3665 Kearny Villa Rd., Suite 200N, San Diego, CA 92123 USA

**Keywords:** Implementation, Implementation leadership, Leadership, Measurement, Confirmatory factory analysis, Evidence-based practice, Behavioral health services

## Abstract

**Background:**

Implementation science has made significant advances in our understanding of organizational factors that impact the implementation process. Critical to those advances has been the development of measures of key implementation-focused organizational constructs, such as implementation leadership. The Implementation Leadership Scale (ILS) was developed to capture leadership behavior identified as critical to implementation effectiveness. Recent research in education has identified additional dimensions of implementation leadership that extend our understanding of how leaders contribute to the successful implementation and sustainment of new practices. The goal of this paper is to validate the extended version of the Implementation Leadership Scale, or the ILS-X, in behavioral health settings.

**Method:**

This paper utilized baseline data from two large implementation trials conducted in behavioral health settings that collected survey data on the ILS-X measure from 389 providers across 68 behavioral health clinics. The ILS-X is a pragmatic measure with 21 items assessing seven dimensions of implementation leadership (proactive, knowledgeable, supportive, perseverant, communication, vision/mission, and available). Analyses assessed internal consistency reliability, interrater reliability and agreement, factor structure, and construct validity evidence for scores on the measure.

**Results:**

The ILS-X performed well across all criteria. A second-order confirmatory factor model fit the data well and had high factor loadings across all dimensions. Correlations with other clinic-level measures (e.g., transformational leadership, organizational climate, aggregate job satisfaction, clinic characteristics) were consistent with theory-guided expectations. Internal consistency reliability and aggregation indices supported future use of the measure.

**Conclusion:**

The ILS-X allows implementation researchers and practitioners to reliably assess a more comprehensive array of implementation leadership behaviors that builds on the original ILS measure. The ILS-X will be valuable for targeting an expanded range of behaviors for identifying areas of leadership strength and improvement during implementation efforts.

**Supplementary Information:**

The online version contains supplementary material available at 10.1186/s43058-025-00786-x.

Contributions to the Literature • Effective implementation is strengthened when leaders support the use of new practices in a variety of ways; these behaviors are referred to as “implementation leadership.”

• Validated measures of implementation leadership can provide information to better understand the influence of leadership on implementation outcomes and to provide feedback to leaders during implementation efforts; however, available measures may not capture the full array of important implementation leadership behaviors in a parsimonious way

• Building on early work on the Implementation Leadership Scale (ILS), this research validates an extended measure of implementation leadership (the ILS-X) that includes additional dimensions, including leaders’ communication, vision/mission, and availability for supporting implementation.

• These new dimensions—along with the prior four dimensions—advance the science of leadership for implementation that can be integrated into extant theories, models, and frameworks and where the roles of leaders and/or champions are deemed important for successful implementation.

## Background

Implementation science has increasingly recognized the importance of the inner-organizational context for implementation effectiveness [1–5]. Although a variety of organizational factors have been studied regarding their influence on implementation processes and outcomes (e.g., organizational climate, organizational culture, readiness for change [5]), perhaps the most common factor discussed in the literature is leadership. Implementation frameworks, such as the Exploration, Preparation, Implementation, and Sustainment (EPIS) model [5], the Consolidated Framework for Implementation Research (CFIR) [3], and the Promoting Action on Research Implementation in Health Services (PARIHS) framework [6], all emphasize how leaders are critical to implementation because of both their direct influence on providers and their indirect influence through shaping organizational structures and organizational climate/culture [7, 8]. For example, a systematic review of EPIS identified the critical role of leadership in both inner (e.g., clinic, organization) and outer (e.g., system, policy) contexts across stages of EBP implementation and sustainment [5]. Accordingly, implementation research has examined how leaders influence a variety of outcomes, including provider attitudes [9], citizenship behavior [10], implementation behavior [8], and implementation outcomes [11]. Additionally, several implementation strategies have been developed that specifically focus on leadership [12–14].

Implementation leadership refers to the behaviors leaders engage in to support the implementation of evidence-based practices (EBPs) [7]. The most common measure of implementation leadership in the literature is the Implementation Leadership Scale (ILS) [15], which captures four dimensions: proactive (i.e., establishes clear standards and planning for EBP implementation), knowledgeable (i.e., is knowledgeable about EBP), supportive (i.e., supports, recognizes and appreciates employee efforts to learn EBP), and perseverant (i.e., perseveres through the challenges of EBP implementation). Originally developed in a mental health context, the ILS has subsequently been validated in other contexts, including substance use treatment [16], child welfare [17], nursing [18], and education [19, 20]. The measure has also been translated into multiple languages, including German [21], Spanish [22], Greek [23], Japanese [24], Norwegian [25], and Chinese [26, 27].

Although most validation studies of the ILS involve minor adaptations to the wording of the original items, recent research by Lyon et al. [20] integrated a multi-stage process of subject matter expert feedback to ensure the dimensions of the ILS were appropriate for school settings and to identify important aspects of implementation leadership not captured by the original ILS dimensions. For the school version of the ILS, the original four dimensions were maintained, and three dimensions were added: communication (“concrete efforts to engage in bidirectional communication surrounding EBP,” p. 10), vision/mission (“how a leader integrates EBP implementation with the core objectives of a school,” p. 10), and availability (“the extent to which leaders are accessible and responsive to staff needs or problems surrounding implementation,” p. 10). Lyon et al. [20] developed items for these additional dimensions and provided evidence for the factorial and construct validity of scores on the adapted measure.

Building on the work of Lyon et al., the goal of this paper was to determine if the expanded version of the ILS could be validated in behavioral health settings where much of the research on implementation leadership has focused. Specifically, we analyzed data from two implementation trials that addressed the role of leaders in implementation and that included the expanded dimensions of the ILS, which we refer to as the ILS-X (or the “extended” ILS). We ran confirmatory factor analyses on these data, in addition to providing construct validity evidence, similar to what was provided for the original ILS. By providing validity evidence for the ILS-X outside of school settings, we hope to provide researchers and practitioners in diverse fields with an instrument covering a broader set of leadership behaviors that can be utilized for understanding and improving the organizational context for implementation, implementation outcomes, and ultimately, client outcomes.

## Method

### Procedures & sample

The data for these analyses were taken from two implementation trials that tested the Leadership and Organizational Change for Implementation (LOCI) strategy on implementation outcomes. The NIMH-funded WISDOM (Working to Implement and Sustain Digital Outcome Measures) trial (R01MH119127) assessed the effect of LOCI on fidelity to measurement-based care and clinical outcomes in youth mental health services across three states in the USA [28]. The NIDA-funded LOCI-SL (LOCI–System Level) trial (R01DA049891) utilized an adapted version of the original LOCI strategy to address the role of the system-level outer context on the implementation of motivational interviewing in adult behavioral health services in one western US state, and implementation of combined motivational enhancement therapy/cognitive behavioral therapy (MET/CBT) in adult and youth behavioral health services in a midwestern US state. Both trials aimed to enhance provider fidelity to the target EBP through engagement of leaders across multiple levels in the LOCI strategy. ILS-X data were collected in both trials via web-based surveys to provide feedback to participating LOCI leaders. Baseline ILS-X data from both trials were used for these analyses. In addition to the ILS-X, measures of general leadership, organizational climate, and job satisfaction were also included to evaluate construct validity.

Data from 389 providers (175 from the WISDOM trial and 214 from the LOCI-SL trial), representing all of providers who completed baseline surveys in the two trials, were utilized in these analyses. Providers were nested within 68 clinics; the number of providers per clinic ranged from 1 to 21, with an average of 5.7 providers per clinic. Sample demographic characteristics can be found in Table 1.

**Table 1:** Sample characteristics

Characteristic	*M* (*n*)	*SD* (*%*)
Age in years – *Mean* (*SD*)	39.36	11.67
Years tenure in organization – *Mean* (*SD*)	3.18	4.38
Highest education level completed – *n (%)*		
GED	5	1.3
High school diploma	4	1.0
Some college	19	4.9
College graduate	61	15.7
Some graduate work	15	3.9
Master’s degree	274	70.4
Doctoral degree or equivalent	11	2.8
Gender identity – *n (%)*		
Female	307	78.9
Male	68	17.5
FTM	1	0.3
Genderqueer	4	1.0
Not disclosed	3	0.8
Prefer to self-identify	6	1.5
Race – *n (%)*		
American Indian/ Alaskan Native	3	0.8
Asian	7	1.8
Black or African American	8	2.1
Native Hawaiian/ Other Pacific Islander	2	0.5
More than one race	15	3.9
Prefer to self-identify	9	2.3
White	332	85.3
Not reported	13	3.3
Ethnicity – *n (%)*		
Hispanic or Latino	44	11.3
Not Hispanic or Latino	342	87.9
Not reported	3	0.8

*N* = 389 providers, *K* = 68 clinics

### Measures

#### Implementation leadership

Implementation leadership was assessed using the original ILS measure [15] plus items assessing the additional three dimensions from the school version of the ILS [20]. Thus, the proposed Implementation Leadership Scale-Extended (or ILS-X) includes 21 items across seven subscales: proactive, knowledgeable, supportive, perseverant, communication, vision/mission, and available (see Additional File 1 for the full scale and scoring instructions). As with the ILS, the ILS-X can be used to assess leadership support for either EBP in general or for specific EBP. In the WISDOM study, the items referred to EBP implementation in general, whereas in the LOCI-SL study, the items were tailored to focus on the specific EBP being implemented. Providers rated their direct supervisor on each item on a scale from 0 (“not at all”) to 4 (“a very great extent”). Mean scores were calculated for each dimension and the total ILS-X.

### Transformational leadership

Transformational leadership was assessed using the Multifactor Leadership Questionnaire (MLQ) [29]. The measure has strong reliability and validity and includes four subscales: idealized influence, inspirational motivation, intellectual stimulation, and individualized consideration. Providers rated their supervisor on a scale from 0 (“not at all”) to 4 (“a very great extent”). Mean scores were calculated for each dimension of transformational leadership and overall transformational leadership.

### Organizational climate

Four dimensions of organizational climate were measured using items from the Organizational Climate Measure (OCM) [30]: involvement, innovation and flexibility, performance feedback, and pressure to produce. Providers responded to items on a scale from 1 (“definitely false”) to 4 (“definitely true”). Mean scores were calculated for each of the four dimensions.

### Job satisfaction

Job satisfaction was measured using the Cammann Job Satisfaction Scale [31]. The measure includes three items that assess the extent to which respondents enjoy their job (from 1 “strongly disagree” to 5 “strongly agree”). Mean scores were calculated for overall job satisfaction.

### Analyses

Reliability of the ILS-X total scale and subscales was evaluated using Cronbach’s alpha coefficient.

The ILS-X was designed to assess providers’ shared perceptions of leadership behaviors, which are typically hypothesized to operate as a unit-level (i.e., clinic-level) construct. As such, scores on the ILS-X should demonstrate (a) homogeneity within clinics, which confirms providers have similar experiences of leadership in their clinics, and (b) variability across clinics, which confirms the leadership experienced by providers clusters in meaningful ways across clinics. Homogeneity of ILS-X ratings within clinics was evaluated using the r_wg(j)_ inter-rater agreement index based on a null distribution [32, 33]. Values of r_wg(j)_ range from 0 to 1 with values > 0.7 representing strong to very strong effect sizes for justifying aggregation [34]. Variability in ILS-X scores across clinics was evaluated using a one-way ANOVA with clinic as the factor, and by examining values of the intraclass correlation coefficient, ICC(1), which quantifies the proportion of variance in provider-level ILS-X scores attributable to clinic membership, with values of 0.10 considered a medium effect size [34].

Confirmatory factor analysis (CFA) was used to generate structural validity evidence for scores on the ILS-X. A second-order CFA model was specified in which items were forced to load on their respective hypothesized subscales and the subscales were forced to load on a single overarching factor representing implementation leadership. To account for clustering of providers within clinics, nonnormal outcomes (e.g., potentially skewed items), and missing data (minimum covariance coverage = 0.99), the model was estimated using the robust maximum likelihood estimator with the TYPE = COMPLEX option in Mplus Version 8.0 [35]. Model fit was evaluated using: (1) root mean square error of approximation (RMSEA), where values < 0.08 demonstrate reasonable fit; (2) comparative fit index (CFI), where values > 0.9 are typically regarded as indicating acceptable fit; and (3) standardized root mean square residual (SRMR), where values < 0.05 are typically accepted as indicative of good fit [36].

Construct validity evidence for scores on the ILS-X total scale and subscales was assessed based on bivariate clinic-level correlations with transformational leadership, organizational climate, job satisfaction, years tenure in organization, and proportion of providers with graduate degrees. The expectation was that correlations with transformational leadership and job satisfaction would be medium-to-high, but lower than the correlations among the ILS dimensions, that the correlations with the organizational climate dimensions would be small-to-medium and lower than the correlations with transformational leadership and job satisfaction, and that the correlations with the aggregate employee characteristics would be near zero.

## Results

### Reliability and aggregation

Table 2 presents values of Cronbach’s coefficient alpha for the ILS-X total score and subscales; all values were in the excellent range (range = 0.92-0.98). Results of the r_wg(j)_ and ICC(1) analyses indicated there was generally strong agreement on ILS-X ratings within clinics and significant variation across clinics. The median r_wg(j)_ value on the ILS-X total score was 0.95 (mean = 0.76, SD = 0.36) which is well above the target of 0.70 to indicate strong agreement and over the target of 0.91 for very strong agreement [34]. Results of the one-way ANOVA indicated significant variation across clinics on ILS-X total score ratings (F = 1.79, df = 67, 320, *p* < 0.001, eta-squared = 0.27) and the ICC(1) of 0.13 indicated a medium effect size [34].

**Table 2:** Descriptive statistics and correlations of Implementation Leadership Scale-Extended (ILS-X) total score, subscales, and original ILS

Scale	Mean	SD	1	2	3	4	5	6	7	8	9
1. ILS-X Total Score	2.53	.59	(.98)								
2. Proactive subscale	1.86	.75	.83	(.92)							
3. Knowledgeable subscale	2.72	.71	.79	.59	(.96)						
4. Supportive subscale	2.80	.58	.91	.68	.75	(.95)					
5. Perseverant subscale	2.65	.67	.92	.70	.73	.93	(.95)				
6. Communication subscale*	2.52	.68	.90	.69	.69	.81	.82	(.93)			
7. Availability subscale*	2.81	.67	.84	.56	.59	.79	.80	.86	(.95)		
8. Vision/mission subscale*	2.37	.72	.92	.79	.69	.81	.84	.92	.75	(.95)	
9. ILS Total Score (original)	2.51	.60	.96	.84	.86	.93	.93	.84	.76	.88	(.97)

*N* = 68 clinics. All correlations are at the clinic level. All correlations are statistically significant at *p* < .001 (two-tailed). Cronbach’s coefficient alpha is reported on the diagonal in parentheses. Subscales marked with an asterisk (*) are new dimensions added to the ILS-X

### Structural validity evidence

**Fig. 1:**
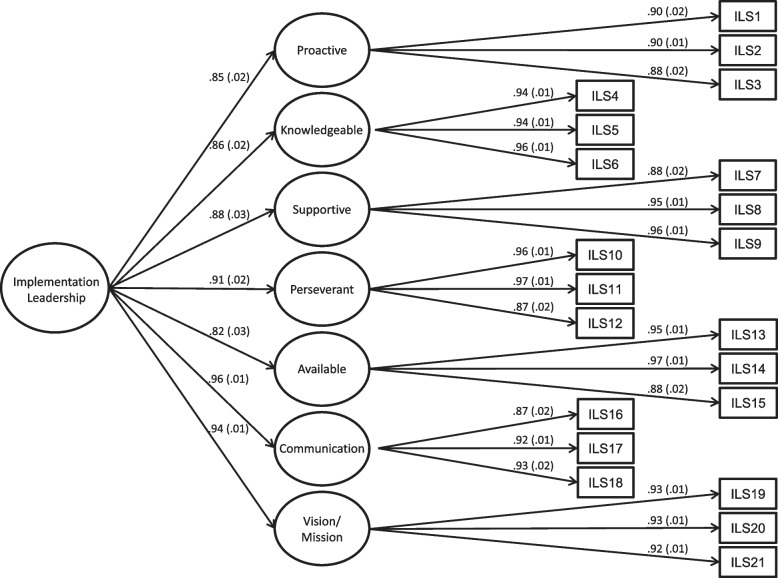
Second order confirmatory factor analysis of Implementation Leadership Scale-Extended (ILS-X). (Note: *N* = 389 providers; K = 68 clinics. Chi-square test of model fit = 564.87, *p* = 0.000; RMSEA = 0.074, 90% CI = 0.067 – 0.080; CFI = 0.944; SRMR = 0.045)

Results of the CFA supported the hypothesized second-order factor structure of the ILS-X (see Fig. 1). All unstandardized factor loadings for the items and subscales were statistically significant at *p* < 0.001. The standardized factor loadings for the items on their respective subscales ranged from 0.88 to 0.97; the standardized loadings for the subscales on the overall factor ranged from 0.82 to 0.96. The model chi-square test did not support the model’s fit to the data (χ^2^ = 564.87, *df* = 182, *p* = 0.000); however, it is known to be biased in large samples. Thus, we relied on results of the other fit indices that indicated acceptable to good model fit (RMSEA = 0.074, 90% CI = 0.067–0.080; CFI = 0.944; SRMR = 0.045). The fit indices along with the strong factor loadings support the hypothesized factor structure of the ILS-X.

### Construct validity evidence

Bivariate clinic-level correlations among the ILS-X total score and subscales are shown in Table 2. The average correlation among the subscales was *r* = 0.75 (*SD* = 0.10, range = 0.56-0.93), indicating they captured distinct but related aspects of implementation leadership. The average correlation between the ILS-X total score and the ILS-X subscales was higher (*r* = 0.87, *SD* = 0.05, range = 0.79-0.92), indicating all of the subscales had a strong relationship with the overall implementation leadership construct.

Results of the bivariate clinic-level correlations between the ILS-X and related variables are shown in Table 3. The pattern of correlations supported the construct validity of scores on the ILS-X. First, the magnitudes of the correlations aligned with expectations. Correlations between the ILS-X and transformational leadership dimensions (average *r* =|.62|) were smaller than the correlations between the ILS-X total score and ILS-X subscale scores (average *r* =|.87|), but greater in magnitude than the correlations between the ILS-X and dimensions of organizational climate (average *r* =|.33|) and between the ILS-X and aggregate job satisfaction (*r* = 0.56). Second, the direction of the correlations aligned with expectations. ILS-X scores were positively correlated with transformational leadership, climate dimensions of employee involvement, innovation and flexibility, and performance feedback, and aggregate job satisfaction; were negatively correlated with the climate dimension of pressure to produce; and had near-zero relationships with aggregate job tenure (*r* = 0.04, *p* = 0.751) and percent of providers with a graduate degree (*r* = 0.03, *p* = 0.790). Overall, these results suggest scores on the ILS-X measure a distinct type of leadership that is related to other organizational characteristics in theoretically meaningful ways.

**Table 3:** Validity correlations

Criterion Variable	Implementation Leadership Scale—Extended (ILS-X)							
	Total Score	Proactive	Knowledgeable	Supportive	Perseverant	Communication	Availability	Vision/ Mission
MLQ Transformational Leadership Scale								
Total Score (*n* = 68)	.65***	.41***	.54***	.64***	.56***	.62***	.61***	.61***
Individualized consideration (*n* = 68)	.58***	.34**	.45***	.58***	.50***	.57***	.61***	.54***
Intellectual stimulation (*n* = 68)	.56***	.40***	.50***	.54***	.43***	.51***	.48***	.50***
Inspirational motivation (*n* = 68)	.61***	.37**	.50***	.61***	.54***	.64***	.59***	.59***
Idealized influence (*n* = 68)	.71***	.45***	.59***	.70***	.68***	.64***	.63***	.68***
Organizational Climate Measure								
Involvement (*n* = 67)	.35**	.13	.41***	.36**	.40***	.29*	.42***	.20
Innovation & flexibility (*n* = 67)	.43***	.27*	.46***	.42***	.46***	.35**	.39**	.31*
Performance feedback (*n* = 67)	.34**	.12	.36**	.32**	.36**	.40***	.43***	.23
Pressure to produce (*n* = 67)	-.19	-.14	-.26*	-.17	-.21	-.08	-.23	-.07
Job Satisfaction (org. mean) (*n* = 67)	.56***	.35**	.57***	.54***	.54***	.48***	.53***	.45***
Years tenure (org. mean) (*n* = 54)	.04	.16	-.03	-.01	.05	.02	.00	.08
% providers with graduate degree (*n* = 68)	.03	.10	.11	.05	.10	-.04	-.04	-.01

All correlations are at the clinic level. Some *n*s are < 68 due to missing data on criterion variables. *MLQ* Multifactor Leadership Questionnaire

^***^ *p* < .001

^**^ *p* < .01

^*^ *p* < .05

## Discussion

The goal of this paper was to evaluate the reliability and validity of scores on an expanded measure of implementation leadership that captured new dimensions of leadership behavior believed to be important for implementation success. Three new dimensions of implementation leadership—communication, vision/mission, and availability—identified through work in schools [20] were evaluated to determine whether they generalized to behavioral health settings. This research not only extends our understanding of implementation leadership, but has broad implications for implementation research, theories, models, and frameworks that invoke leadership as an important construct for implementation science [3, 5, 37, 38].

The results for the extended version of the ILS (or ILS-X) were very positive. The individual dimensions had very high internal consistency despite only having three items, and showed support for aggregating overall ILS-X scores to the clinic level. The fit for the CFA models indicated strong support for the structural validity of scores on the ILS-X. This indicates that the proposed items assess a shared underlying construct (i.e., implementation leadership) comprised of the proposed dimensions, as evidenced by the concordance of the hypothesized statistical model of relationships and the observed data. Finally, the pattern of correlations with other measures (i.e., transformational leadership, job satisfaction, organizational climate, and employee characteristics) were as expected and provided strong construct validity evidence for scores on the measure.

There are several notable results in the analyses. Of the transformational leadership dimensions, the ILS-X total score was most highly correlated with idealized influence. Idealized influence represents leaders as role models who discuss their core values and ethics [39]. Thus, there appears to be a connection between leaders’ support of implementation efforts and being viewed as individuals worth emulating who stand up for their beliefs. The organizational climate dimension most highly correlated with the ILS-X was innovation and flexibility. This finding is consistent with the idea that the implementation of new practices is innovative and connected to the organization’s goal to integrate new research and norms in the field. Finally, the strong correlations between the ILS-X dimensions and aggregate job satisfaction suggests that employees working for supervisors high in implementation leadership are more satisfied and engaged in their jobs, perhaps because of the clear goals they communicate relative to leaders more ambivalent about implementation efforts.

It is worth acknowledging the pros and cons of using the ILS-X versus the original version of the ILS. The original ILS is 12 items, a shorter measure relative to the 21-item ILS-X, and the original ILS has been validated in a variety of contexts and in a variety of languages. Thus, the original ILS may be preferred in many circumstances, especially when respondent time is limited. However, there are benefits to the longer ILS-X. Because the ILS-X captures a wider variety of leader behaviors, feedback to leaders using the ILS-X would include more ideas for improving their leadership; in other words, it gives them more tools in their leadership toolkit. The correlation between the total score based on the original ILS dimensions and the ILS-X total score including the additional dimensions was 0.96 in this study, suggesting that both are capturing the same underlying construct. In practical terms, it means that leaders who perform the behaviors captured by the original ILS are likely to perform the new behaviors addressed in the ILS-X. Thus, researchers or practitioners who have limited space may opt for the original instrument, whereas applications focused on leadership feedback and development [40] may prefer the longer ILS-X to provide a more well-rounded perspective on how leaders can impact implementation effectiveness.

There are a variety of opportunities for future research to build on results presented here. For instance, future research could examine the invariance of the instrument in additional settings and populations, and could evaluate the role of leadership across phases of implementation. Future research could also examine the relationships between implementation leadership as measured by the ILS-X and measures of other organizational constructs, such as the implementation climate scale [41] and the organizational readiness change assessment [42]. The validation of the ILS-X also opens up a variety of opportunities for leadership development within healthcare organizations. For instance, measurement and feedback is already a critical piece of leadership-focused implementation interventions like LOCI; the ILS-X expands our understanding of the critical behaviors leaders can be encouraged to perform to enhance implementation efforts. The ILS could also be linked to specific implementation strategies, such as those discussed in ERIC [43] to enhance intervention planning and leadership support for implementation.

## Conclusion

In summary, there was strong evidence for the validity of the ILS-X and the additional implementation leadership dimensions identified by Lyon et al. [20] in behavioral health settings. Researchers should continue to investigate the outcomes associated with implementation leadership in other settings and how tools like the ILS-X can provide practical insights to leaders to improve their own approach to implementation and the implementation outcomes of their units.

## Supplementary Information

Additional File 1. Title: Implementation Leadership Scale-Extended (ILS-X). Description: ILS-X items with scoring instructions.

## Data Availability

The datasets analyzed during the current study are available from the corresponding author on reasonable request.

## Abbreviations

ANOVA: Analysis of variance
CFA: Confirmatory factor analysis
CFI: Comparative fit index
EBP: Evidence-based practice
ICC: Intraclass correlation
ILS: Implementation Leadership Scale
ILS-X: Implementation Leadership Scale – Extended
LOCI: Leadership and Organizational Change for Implementation
LOCI-SL: Leadership and Organizational Change for Implementation – System Level
MET/CBT: Motivational enhancement therapy/cognitive behavioral therapy
MLQ: Multifactor Leadership Questionnaire
NIDA: National Institute on Drug Abuse
NIMH: National Institude of Mental Health
OCM: Organizational Climate Measure
RMSEA: Root mean square error of approximation
SRMR: Standardized root mean square residual
WISDOM: Working to Implement and Sustain Digital Outcome Measures

## References

[1] Aarons GA, Ehrhart MG, Lengnick-Hall R, Moullin JC. The role of organizational processes in dissemination and implementation research. In: Oxford: Oxford University Press. Dissemination and implementation research in health: Translating science to practice. 3^rd^ ed. 2023:192–211.

[2] Allen JD, Towne SD, Maxwell AE, DiMartino L, Leyva B, Bowen DJ, Linnan L, Weiner BJ. Measures of organizational characteristics associated with adoption and/or implementation of innovations: a systematic review. BMC Health Services Res. 2017;17:591. 10.1186/s12913-017-2459-x PMC5569532 28835273

[3] Damschroder LJ, Reardon CM, Widerquist MAO, Lowery J. The updated Consolidated Framework for Implementation Research based on user feedback. Implement Sci. 2022;17(1):75. 36309746 10.1186/s13012-022-01245-0 PMC9617234

[4] Glisson C, Williams NJ. Assessing and changing organizational social contexts for effective mental health services. Annu Rev Public Health. 2015;36:507–23. 25785894 10.1146/annurev-publhealth-031914-122435

[5] Moullin JC, Dickson KS, Stadnick NA, Rabin B, Aarons GA. Systematic review of the exploration, preparation, implementation, sustainment (EPIS) framework. Implement Sci. 2019. 10.1186/s13012-018-0842-6. 10.1186/s13012-018-0842-6 PMC6321673 30611302

[6] Rycroft-Malone J. The PARIHS framework—a framework for guiding the implementation of evidence-based practice. J Nurs Care Qual. 2004;19(4):297–304. 15535533 10.1097/00001786-200410000-00002

[7] Aarons GA, Ehrhart MG, Farahnak LR, Sklar M. Aligning leadership across systems and organizations to develop a strategic climate for evidence-based practice implementation. Annu Rev Public Health. 2014;35:255–74. 24641560 10.1146/annurev-publhealth-032013-182447 PMC4348088

[8] Williams NJ, Wolk CB, Becker-Haimes EM, Beidas RS. Testing a theory of strategic implementation leadership, implementation climate, and clinicians’ use of evidence-based practice: a 5-year panel analysis. Implementation Science. 2020;15(10). 10.1186/s13012-020-0970-7 PMC7006179 32033575

[9] Powell BJ, Beidas RS, Lewis CC, Aarons GA, McMillen JC, Proctor EK, et al. Methods to improve the selection and tailoring of implementation strategies. J Behav Health Serv Res. 2017;44:177–94. 26289563 10.1007/s11414-015-9475-6 PMC4761530

[10] Borge RH, Skar AMS, Endsjø M, Egeland KM. Good soldiers in implementation: Validation of the implementation citizenship behavior scale and its relation to implementation leadership and intentions to use evidence-based practices. Implementation Science Communications. 2021;2(1):136. 10.1186/s43058-021-00240-8 PMC8665530 34895347

[11] Williams NJ, Hugh ML, Cooney DJ, Worley JA, Locke J. Testing a theory of implementation leadership and climate across autism evidence-based interventions of varying complexity. Behav Ther. 2022;53:900–12. 35987547 10.1016/j.beth.2022.03.001 PMC9395730

[12] Aarons GA, Ehrhart MG, Moullin JC, Torres EM, Green AE. Testing the leadership and organizational change for implementation (LOCI) intervention in substance abuse treatment: A cluster randomized trial study protocol. Implementation Science. 2017;12(1):29. 10.1186/s13012-017-0562-3 PMC5335741 28253900

[13] Gifford W, Lewis KB, Eldh AC, Fiset V, Abdul-Fatah T, Aberg AC, Thavorn K, Graham ID, Wallin L. Feasibility and usefulness of a leadership intervention to implement evidence-based falls prevention practices in residential care in Canada. Pilot Feasibility Studies. 2019;5(1):103. 31452925 10.1186/s40814-019-0485-7 PMC6701101

[14] Richter A, Lornudd C, von Thiele Schwarz U, Lundmark R, Mosson R, Skoger UE, et al. Evaluation of iLead, a generic implementation leadership intervention: mixed-method preintervention-postintervention design. BMJ Open. 2020. 10.1136/bmjopen-2019-033227. 10.1136/bmjopen-2019-033227 PMC7045007 31932392

[15] Aarons GA, Ehrhart MG, Farahnak LR. The implementation leadership scale (ILS). Development of a brief measure of unit level implementation leadership. Implement Sci. 2014;9(1):45. 10.1186/1748-5908-9-45 PMC4022333 24731295

[16] Aarons GA, Ehrhart MG, Torres EM, Finn NK, Roesch SC. Validation of the implementation leadership scale (ILS) in substance use disorder treatment organizations. J Subst Abuse Treat. 2016;68:31–5. 27431044 10.1016/j.jsat.2016.05.004 PMC5349507

[17] Finn NK, Torres EM, Ehrhart MG, Roesch SC, Aarons GA. Cross-validation of the implementation leadership scale (ILS) in child welfare service organizations. Child Maltreat. 2016;21:250–5. 27002137 10.1177/1077559516638768

[18] Shuman CJ, Ehrhart MG, Torres EM, Veliz P, Kath LM, VanAntwerp K, et al. EBP implementation leadership of frontline nurse managers: validation of the implementation leadership scale in acute care. Worldviews Evid Based Nurs. 2019;17(1):82–91. 31638315 10.1111/wvn.12402

[19] Lyon AR, Cook CR, Brown EC, Locke J, Davis C, Ehrhart MG, Aarons GA. Assessing organizational implementation context in the education sector: Confirmatory factor analysis of measures of implementation leadership, climate and citizenship. Implement Sci. 2018;13(1):5. 29310673 10.1186/s13012-017-0705-6 PMC5759223

[20] Lyon AR, Corbin CM, Brown EC, Ehrhart MG, Locke J, Davis C, et al. Leading the charge in the education sector: Development and validation of the School Implementation Leadership Scale (SILS). Implement Sci. 2022. 10.1186/s13012-022-01222-7. 10.1186/s13012-022-01222-7 PMC9295535 35854385

[21] Söling S, Pfaff H, Karbach U, Ansmann L, Köberlein-Neu J. How is leadership behavior associated with organization-related variables? Translation and psychometric evaluation of the implementation leadership scale in German primary healthcare. BMC Health Services Res. 2022;22(1):1065. 10.1186/s12913-022-08434-z PMC9391066 35986273

[22] Llarena M, Rogers HL, Macia P, Pablo S, Gonzalez-Saenz de Tejada M, Montejo M, Paniagua N, Benito J, Rueda M, Santos B, Grandes G, Sanchez A. Validity and reliability of the transculturally adapted Spanish version of the Implementation Leadership Scale (ILS). Implementation Science Communications. 2023;4(1):112. 10.1186/s43058-023-00495-3 PMC10496227 37700388

[23] Mandrou E, Tsounis A, Sarafis P. Validity and reliability of the Greek version of implementation leadership scale. BMC Psychol. 2020;8(49):1–7. 32410660 10.1186/s40359-020-00413-5 PMC7226931

[24] Saiki M, Tomotaki A, Fukahori H, Yamamoto T, Nishigaki M, Matsuoka C, et al. Reliability and validity of the Japanese version of the Implementation Leadership Scale for nurse managers and staff nurses: a cross-sectional study. J Nurs Manag. 2023. 10.1155/2023/4080434. 10.1155/2023/4080434 PMC11918985 40225600

[25] Braathu N, Laukvik EH, Egeland KM, Skar AMS. Validation of the Norwegian versions of the Implementation Leadership Scale (ILS) and Multifactor Leadership Questionnaire (MLQ) in a mental health care setting. BMC Psychol. 2022;10(1):25. 35135616 10.1186/s40359-022-00725-8 PMC8822706

[26] Hu J, Gifford W, Ruan H, Harrison D, Li Q, Ehrhart MG, et al. Translation and linguistic validation of the implementation leadership scale in Chinese nursing context. J Nurs Manag. 2019;27(5):1030–8. 30861240 10.1111/jonm.12768

[27] Hu J, Gifford W, Ruan H, Harrison D, Li Q, Ehrhart MG, et al. Validating the implementation in Chinese nursing context: a cross-sectional study. Nurs Open. 2021;8(6):3420–9. 33960677 10.1002/nop2.888 PMC8510775

[28] Williams NJ, Marcus SC, Ehrhart MG, Sklar M, Esp S, Carandang K, et al. Randomized trial of an organizational implementation strategy to improve measurement-based care fidelity and youth outcomes in community mental health. J Am Acad Child Adolesc Psychiatry. 2024;63(10):991–1004. 38070868 10.1016/j.jaac.2023.11.010 PMC11265517

[29] Bass BM, Avolio BJ. Multifactor leadership questionnaire. Western J Nursing Res. 1995. 10.1037/t03624-000.

[30] Patterson MG, West MA, Shackleton VJ, Dawson JF, Lawthom R, Maitlis S, et al. Validating the organizational climate measure: links to managerial practices, productivity and innovation. J Organ Behav. 2005;26(4):379–408.

[31] Cammann C, Fichman M, Jenkins GD, Klesh JR. Assessing the attitudes and perceptions of organizational members. In: Hoboken: John Wiley & Sons. Assessing organizational change: A guide to methods, measures, and practices. 1983:71–138.

[32] James LR, Demaree RG, Wolf G. An assessment of within-group interrater agreement. J Appl Psychol. 1993;78(2):306–9.

[33] Lengnick-Hall R, Williams NJ, Ehrhart MG, Willging C, Bunger A, Beidas R, Aarons GA. Eight characteristics of rigorous multilevel implementation research: A step-by-step guide. Implementation Science 202;18(1):52. 10.1186/s13012-023-01302-2 PMC10594828 37872618

[34] LeBreton JM, Senter JL. Answers to 20 questions about interrater reliability and interrater agreement. Organ Res Methods. 2008;11(4):815–52.

[35] Muthén LK, Muthén BO. Mplus user’s guide. 8^th^ ed. Muthén & Muthén. 1998–2017.

[36] Schreiber JB, Nora A, Stage FK, Barlow EA, King J. Reporting structural equation modeling and confirmatory factor analysis results: a review. J Educ Res. 2006;99(6):323–38.

[37] Aarons GA, Sommerfeld DH, Willging CE. The soft underbelly of system change: the role of leadership and organizational climate in turnover during statewide behavioral health reform. Psychol Serv. 2011;8(4):269. 22229021 10.1037/a002619 PMC3252234

[38] Feldstein AC, Glasgow RE. A practical, robust implementation and sustainability model (PRISM) for integrating research findings into practice. Jt Comm J Qual Patient Saf. 2008;34(4):228–43. 18468362 10.1016/s1553-7250(08)34030-6

[39] Bass BM. Does the transactional-transformational leadership paradigm transcend organizational and national boundaries? Am Psychol. 1997;52(4):130–9.

[40] Sklar M, Ehrhart MG, Ramirez N, Carandang K, Kuhn N, Day A, et al. Implementation leadership and implementation climate in context: a single organization intrinsic case study for implementation of digital measurement-based care. Implement Res Pract. 2024;5:1–14. 10.1177/26334895241236680 PMC10976481 38550748

[41] Ehrhart MG, Aarons GA, Farahnak LR. Assessing the organizational context for EBP implementation: the development and validity testing of the implementation climate scale (ICS). Implement Sci. 2014;9:157. 25338781 10.1186/s13012-014-0157-1 PMC4210525

[42] Helfrich CD, Li YF, Sharp ND, Sales AE. Organizational readiness to change assessment (ORCA): development of an instrument based on the promoting action on research in health services (PARIHS) framework. Implement Sci. 2009;4(38):1–13. 19594942 10.1186/1748-5908-4-38 PMC2716295

[43] Powell BJ, Waltz TJ, Chinman MJ, Damschroder LJ, Smith JL, Matthieu MM, et al. A refined compilation of implementation strategies: results from the expert recommendations for implementing change (ERIC) project. Implement Sci. 2015;10(21):1–4. 25889199 10.1186/s13012-015-0209-1 PMC4328074

