# Supplementary material for: Validation of the extended version of the Implementation Leadership Scale (ILS-X)
Source: Implement Sci Commun. 2025 Dec 4;6:133. doi: 10.1186/s43058-025-00786-x (PMC12676754; doi:10.1186/s43058-025-00786-x)
Supplement: Supplementary file 1 — Additional File 1. Title: Implementation Leadership Scale-Extended (ILS-X). Description: ILS-X items with scoring instructions. [file 43058_2025_786_MOESM1_ESM.pdf]

Implementation Leadership Scale – Extended (ILS-X)

The ILS-X measures the behaviors leaders engage in to support the implementation of evidence-based practices (EBPs). There are seven dimensions within the ILS-X measure: proactive, knowledgeable, supportive, perseverant, communication, availability, and vision/mission. In addition to the staff version of the ILS-X, which is used for staff to rate their supervisor/leader and which was validated in this manuscript, we also include a version for supervisors/leaders to report about themselves and one for executives or upper-level leaders to rate supervisors/leaders who report to them, as these may be helpful for the use of the measure in future research and practice. However, as of the publication of the associated manuscript, these additional versions have not been validated.

Scoring:

- The score for each dimension is created by computing a mean score for each set of items that load that dimension.
- A mean of the dimension scores may be computed to yield the mean score for the total ILS-X.

Additional notes:

- It is recommended that the actual name of the supervisor be inserted into the measure to ensure that staff are rating the correct individual. However, a generic referent of "my supervisor" can also be used.
- The terms used for individual workers in this version include "staff" and "team," as well as "agency" when referring to the organization in general. These terms can be adapted to fit the relevant context.
- This version of the measure refers to general EBP. However, the measure can also be used to focus on specific EBP by replacing "evidence-based practice" with the specific name of the practice being implemented.

For further information, contact Dr. Mark Ehrhart at mark.ehrhart@ucf.edu
or Dr. Gregory Aarons at gaarons@health.ucsd.edu.

Staff Version

Please indicate the extent to which you agree with each statement.

0<br>Not at all	1<br>Slight extent	2<br>Moderate extent	3<br>Great extent	4<br>Very great extent

Proactive

1. [Name of Supervisor] has developed a plan to facilitate implementation of evidence-based practice 0 1 2 3 4

2. [Name of Supervisor] has removed obstacles to the implementation of evidence-based practice 0 1 2 3 4

3. [Name of Supervisor] has established clear department standards for the implementation of evidence-based practice 0 1 2 3 4

Knowledgeable

4. [Name of Supervisor] is knowledgeable about evidence-based practice 0 1 2 3 4

5. [Name of Supervisor] is able to answer my questions about evidence-based practice 0 1 2 3 4

6. [Name of Supervisor] knows what they are talking about when it comes to evidence-based practice 0 1 2 3 4

Supportive

7. [Name of Supervisor] recognizes and appreciates employee efforts toward successful implementation of evidence-based practice 0 1 2 3 4

8. [Name of Supervisor] supports employee efforts to learn more about evidence-based practice ... 0 1 2 3 4

9. [Name of Supervisor] supports employee efforts to use evidence-based practice 0 1 2 3 4

Perseverant

10. [Name of Supervisor] perseveres through the ups and downs of implementing evidence-based practice 0 1 2 3 4

11. [Name of Supervisor] carries on through the challenges of implementing evidence-based practice 0 1 2 3 4

12. [Name of Supervisor] reacts to critical issues regarding the implementation of evidence-based practice by openly and effectively addressing the problem(s) 0 1 2 3 4

Available

13. [Name of Supervisor] is accessible if I need help with implementing evidence-based practice 0 1 2 3 4

14. [Name of Supervisor] is available to discuss evidence-based practice 0 1 2 3 4

15. If I have a problem or concern regarding evidence-based practice, I can contact [Name of Supervisor] 0 1 2 3 4

Communication

16. [Name of Supervisor] establishes clear communication systems about evidence-based practice 0 1 2 3 4

17. [Name of Supervisor] talks about evidence-based practice... 0 1 2 3 4

18. [Name of Supervisor] encourages others to communicate with them about evidence-based practice 0 1 2 3 4

Vision/Mission

19. [Name of Supervisor] links the implementation of evidence-based practice to improved client outcomes.. 0 1 2 3 4

20. [Name of Supervisor] has a clear vision for the implementation of evidence-based practice...... 0 1 2 3 4

21. [Name of Supervisor] connects evidence-based practice to the broader mission of our agency 0 1 2 3 4

Supervisor Version (Self-Ratings)

(NOTE: As of the date of the publication of the associated manuscript, this version has not been validated in empirical research)

Please indicate the extent to which you agree with each statement.

0	1	2	3	4
**Not at all**	**Slight extent**	**Moderate extent**	**Great extent**	**Very great extent**

Proactive

1. I have developed a plan to facilitate implementation of evidence-based practice........................ 0 1 2 3 4

2. I have removed obstacles to the implementation of evidence-based practice............................. 0 1 2 3 4

3. I have established clear department standards for the implementation of evidence-based practice.. 0 1 2 3 4

Knowledgeable

4. I am knowledgeable about evidence-based practice 0 1 2 3 4

5. I am able to answer staff's questions about evidence-based practice 0 1 2 3 4

6. I know what I am talking about when it comes to evidence-based practice 0 1 2 3 4

Supportive

7. I recognize and appreciate employee efforts toward successful implementation of evidence-based practice 0 1 2 3 4

8. I support employee efforts to learn more about evidence-based practice.................................. 0 1 2 3 4

9. I support employee efforts to use evidence-based practice 0 1 2 3 4

Perseverant

10. I persevere through the ups and downs of implementing evidence-based practice 0 1 2 3 4

11. I carry on through the challenges of implementing evidence-based practice 0 1 2 3 4

12. I react to critical issues regarding the implementation of evidence-based practice by openly and effectively addressing the problem(s)... 0 1 2 3 4

Available

13. I am accessible if staff need help with implementing evidence-based practice 0 1 2 3 4

14. I am available to discuss evidence-based practice... 0 1 2 3 4

15. If they have a problem or concern regarding evidence-based practice, staff can contact me 0 1 2 3 4

Communication

16. I establish clear communication systems about evidence-based practice.................................... 0 1 2 3 4

17. I talk about evidence-based practice.. 0 1 2 3 4

18. I encourage others to communicate with me about evidence-based practice implementation 0 1 2 3 4

Vision/Mission

19. I link the implementation of evidence-based practice to improved client outcomes 0 1 2 3 4

20. I have a clear vision for the implementation of evidence-based practice in this team 0 1 2 3 4

21. I connect evidence-based practice to the broader mission of our agency 0 1 2 3 4

Executive Version

(NOTE: As of the date of the publication of the associated manuscript, this version has not been validated in empirical research)

Please indicate the extent to which you agree with each statement.

0<br>Not at all	1<br>Slight extent	2<br>Moderate extent	3<br>Great extent	4<br>Very great extent

Proactive

1. [Name of Supervisor] developed a plan to facilitate implementation of evidence-based practice 0 1 2 3 4

2. [Name of Supervisor] has removed obstacles to the implementation of evidence-based practice 0 1 2 3 4

3. [Name of Supervisor] has established clear department standards for the implementation of evidence-based practice 0 1 2 3 4

Knowledgeable

4. [Name of Supervisor] is knowledgeable about evidence-based practice 0 1 2 3 4

5. [Name of Supervisor] is able to answer staff's questions about evidence-based practice 0 1 2 3 4

6. [Name of Supervisor] knows what they are talking about when it comes to evidence-based practice 0 1 2 3 4

Supportive

7. [Name of Supervisor] recognizes and appreciates employee efforts toward successful implementation of evidence-based practice 0 1 2 3 4

8. [Name of Supervisor] supports employee efforts to learn more about evidence-based practice ... 0 1 2 3 4

9. [Name of Supervisor] supports employee efforts to use evidence-based practice 0 1 2 3 4

Perseverant

10. [Name of Supervisor] perseveres through the ups and downs of implementing evidence-based practice 0 1 2 3 4

11. [Name of Supervisor] carries on through the challenges of implementing evidence-based practice 0 1 2 3 4

12. [Name of Supervisor] reacts to critical issues regarding the implementation of evidence-based practice by openly and effectively addressing the problem(s) 0 1 2 3 4

Available

13. [Name of Supervisor] is accessible if staff need help with implementing evidence-based practice 0 1 2 3 4

14. [Name of Supervisor] is available to discuss evidence-based practice 0 1 2 3 4

15. If they have a problem or concern regarding evidence-based practice, staff can contact [Name of Supervisor] 0 1 2 3 4

Communication

16. [Name of Supervisor] establishes clear communication systems about evidence-based practice 0 1 2 3 4

17. [Name of Supervisor] talks about evidence-based practice........ 0 1 2 3 4

18. [Name of Supervisor] encourages others to communicate with them about evidence-based practice 0 1 2 3 4

Vision/Mission

19. [Name of Supervisor] links the implementation of evidence-based practice to improved client outcomes........ 0 1 2 3 4

20. [Name of Supervisor] has a clear vision for the implementation of evidence-based practice...... 0 1 2 3 4

21. [Name of Supervisor] connects evidence-based practice to the broader mission of our agency 0 1 2 3 4
